# Supplementary material for: Genotypic and Phenotypic Characterization of Pseudomonas atacamensis EMP42 a PGPR Strain Obtained from the Rhizosphere of Echinocactus platyacanthus (Sweet Barrel)
Source: Microorganisms. 2024 Jul 24;12(8):1512. doi: 10.3390/microorganisms12081512 (PMC11356332; doi:10.3390/microorganisms12081512)
Supplement: Supplementary file 1 [file microorganisms-12-01512-s001.zip › Figure S1_Pan-Core curve.pdf]

(A)

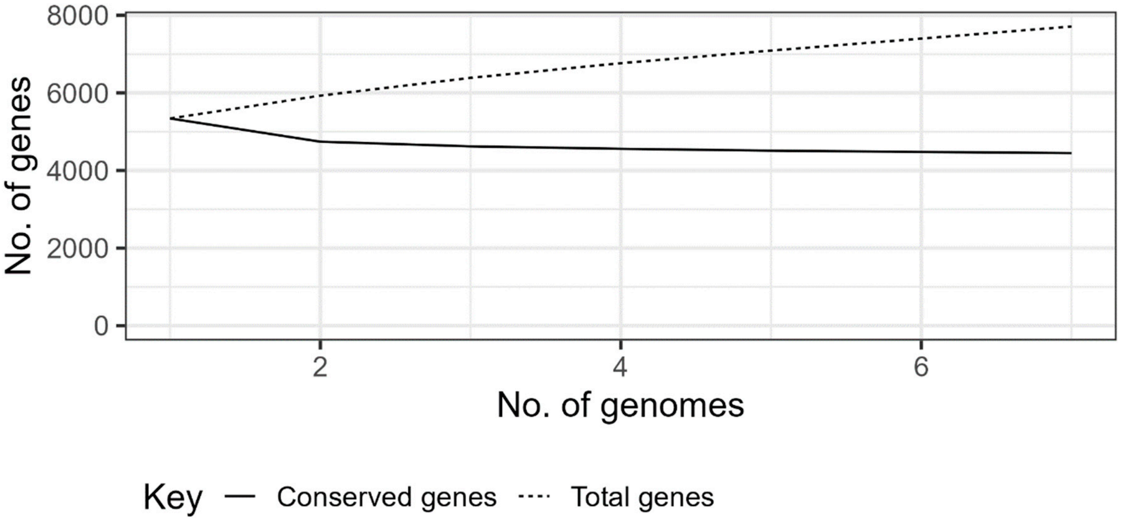

(B)

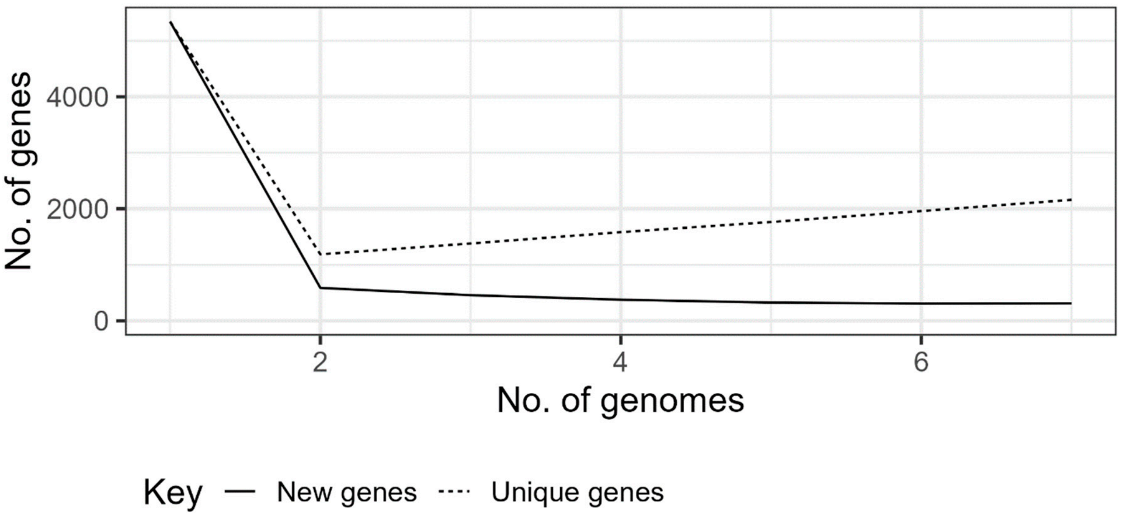

Figure S1: *Pan-Core* curve. The pan genome of *P. atacamensis* varies as genomes are randomly added. The ascending dotted lines are typical of open pangenomes. (A) Comparison of conserved genes vs total genes added to the genomes. (B) Comparison new genes vs unique genes added to the genomes.
